# The Management of Ischemic Priapism Due to Sickle Cell Disease and Other Etiologies: Treatment Strategies and Indications for Penile Prosthesis Implantation in an Endemic Region

**DOI:** 10.3390/medicina61040658

**Published:** 2025-04-03

**Authors:** Eser Ördek, Sadık Görür, Fatih Gökalp, Duran Kuru, Ferhat Uçurmak

**Affiliations:** Department of Urology, Faculty of Medicine, Hatay Mustafa Kemal University, Hatay 31060, Turkey; sadikgorur@gmail.com (S.G.); fatihgokalp85@gmail.com (F.G.); durankuru94@gmail.com (D.K.); f.ucurmak@gmail.com (F.U.)

**Keywords:** priapism, sickle cell disease, penile prosthesis

## Abstract

*Background and Objectives*: Priapism is a condition characterized by a prolonged erection lasting over four hours, either independent of or following sexual stimulation. The primary treatment goal for ischemic and non-ischemic priapism is timely and appropriate intervention in order to preserve erectile function and penile length. This study aims to evaluate the management of recurrent ischemic priapism in sickle cell disease (SCD) patients in an endemic region and compare it with ischemic priapism of other etiologies. *Materials and Methods*: Patients admitted to our hospital with a diagnosis of priapism between January 2010 and June 2024 were retrospectively reviewed. The patients were divided into two groups: ischemic priapism due to SCD and ischemic priapism due to other etiologies. Patient characteristics, treatment management, and the need for penile prosthesis (PP) were compared. *Results*: A total of 40 ischemic priapism patients were included in the study; 20 of them had SCD and the other 20 had priapism cases due to different etiologies. In the SCD priapism group, the rate of comorbidity and previous history of priapism were significantly (*p* < 0.05) higher than in the other etiologies of priapism group. Similarly, in the SCD priapism group, the hospital admission time and the rate of fibrosis findings in MRI (magnetic resonance imaging) were significantly (*p* < 0.05) higher than in the other priapism groups. The PP implantation rate in the SCD priapism group was found to be significantly (*p* < 0.05) higher than in the other priapism group. *Conclusions*: This study highlights the importance of early intervention and patient awareness in SCD-related ischemic priapism, recommending educational programs to improve symptom recognition and prevent complications.

## 1. Introduction

Priapism is a penile erection disorder characterized by an erection that persists or occurs independently of sexual desire or stimulation [1]. Priapism is defined as involuntary erections that usually last for 4 h or more. Priapism can be a disabling condition that can significantly affect the quality of life of those affected, both physically and psychologically. Although it can occur at any age, its incidence in the general population is low, with a rate of 0.5 to 0.9 cases per 100,000 person-years [2]. In the inherited disease SCD, its prevalence can be as high as 42% [3]. Recent studies have also highlighted the significant psychological distress experienced by patients with recurrent priapism, particularly those with SCD, emphasizing the need for early intervention and comprehensive care. Priapism is divided into three main types: ischemic, non-ischemic and recurrent ischemic priapism [4]. Ischemic priapism is the most common type, accounting for 95% of all cases [5].

Although most episodes of priapism resolve spontaneously, those lasting longer than 4 h are considered urological emergencies and require immediate intervention [6]. The severity of ischemic priapism is related to the duration of the erection and the extent of tissue ischemia, which leads to irreversible damage if not treated promptly. The primary goal of treatment is to manage pain, reduce cavernosal pressure, and restore arterial blood flow to minimize the risk of long-term complications resulting from erectile tissue damage [7]. Several treatment modalities can be used to achieve this goal: first-line treatments include cavernosal aspiration, normal saline irrigation, intracavernosal injections, and surgical shunting [8]. In cases where conservative treatment and shunts fail, the early placement of a PP is one of the best options to preserve erectile function and prevent corporal fibrosis [9]. The American Urological Association (AUA) recommends urgent PP implantation in cases lasting longer than 36 h and its use as a first-line treatment in cases lasting longer than 72 h [10].

Ischemic priapism, affecting about 33% of SCD patients, is a frequent yet often neglected complication [11]. The pathophysiology of priapism in SCD is complex and involves vaso-occlusion in the penile microcirculation leading to ischemia and impaired venous outflow. Approximately 74% of priapism episodes in adult men with SCD recur, with 95% of these episodes being ischemic and painful [6]. Neglecting or delaying the treatment of priapism in these patients can result in permanent tissue damage and erectile dysfunction [12]. In addition, recurrent priapism in SCD patients is associated with a higher incidence of corporal fibrosis, further complicating management and outcomes. In this study, we conducted a comparative analysis of recurrent ischemic priapism and its management in SCD patients in an endemic region, and compared it with cases of ischemic priapism of other etiologies.

## 2. Materials and Methods

A retrospective review of medical records was conducted on 40 adult male patients diagnosed with ischemic priapism in the emergency department and urology outpatient clinic of our institution between January 2014 and June 2024. The study was conducted in accordance with the Declaration of Helsinki and approved by the Ethics Committee of Hatay Mustafa Kemal University (approval number: 03/09/2024-32), and all patients provided written informed consent. Patients younger than 18 years old and older than 65 years old, those with non-ischemic etiology or shuttering priapism, or patients who had history of erectile dysfunction treatment such as intracavernosal injection were excluded due to the risk of cavernosal fibrosis. However, we did not exclude patients receiving PDE5 inhibitors. In addition, in cases where differentiation was necessary, cavernosal blood gas analysis was performed considering blood color, pH, pO_2_, and pCO_2_ levels according to standard diagnostic criteria. The cohort was divided into two groups: Group 1 included patients with low-flow (ischemic) priapism due to etiologies other than SCD, while Group 2 included patients with SCD-associated low-flow (ischemic) priapism.

We confirmed that all the included patients had a documented history of priapism and were diagnosed through clinical examination, imaging, and blood gas analysis, following established diagnostic protocols. A comparative analysis was performed between the two groups to assess factors such as the presenting symptoms, duration of erections, history of priapism, medication adherence in SCD patients, treatment modalities, and complications. Patient data on comorbidities, family history of priapism, and previous treatments were also reviewed to identify any confounding factors that might influence treatment outcomes. Comorbidities were present in only two patients, one of hypertension and one of asthma; we did not exclude these patients to avoid heterogeneity. Early postoperative patient satisfaction was evaluated within three months based on medical records and categorized into three levels: “very satisfied”, “satisfied”, and “not satisfied”.

After being informed of the treatment procedures, patients provided written informed consent. Patients received intravenous ceftriaxone as antibiotic prophylaxis prior to surgery. Intracavernosal injections and saline irrigation were used as initial treatments in all patients, with follow-up evaluations to assess the need for further interventions such as shunt surgery or PP implantation. Cavernosal aspiration and saline irrigation were performed under sterile conditions for the initial diagnosis and treatment. Blood gas analysis was performed on the aspirated bodily blood. Patients who did not respond to aspiration were treated with intracorporeal sympathomimetic injection or surgical shunting. Patients who did not respond to initial treatment within 36–48 h underwent penile MRI or Doppler ultrasound. Ultrasound supported low-flow (ischemic) priapism; if the MRI findings confirmed fibrosis, such as corporal scarring or loss of cavernosal tissue elasticity, we offered the patient PP implantation. With the patients’ consent, PP implantation was carried out under general anesthesia using either a malleable or an inflatable prosthesis.

### Statistical Analysis

Descriptive statistics including mean, standard deviation, median, minimum, maximum, frequency, and proportion were used to summarize the data. The Kolmogorov–Smirnov and Shapiro–Wilk tests were employed to assess the normality of the data distribution. The independent sample *t* test was used to analyze normally distributed quantitative independent variables. For non-normally distributed quantitative independent variables, the Mann–Whitney U test was applied. The chi-square test was used to analyze categorical independent variables, and Fisher’s exact test was used when the chi-square test assumptions were not met. All analyses were performed using SPSS version 27.0.

## 3. Results

A total of 40 male patients were included in the study. The mean age of the patients in the non-SCD priapism group (Group 1) was 42.5 years, while in the SCD group (Group 2) it was 40 years, with no significant difference (*p* > 0.05). There were no significant differences between the two priapism groups in terms of smoking, pre-PPI aspiration, and history of erectile dysfunction (ED) (*p* > 0.05). However, the rate of comorbidities and history of priapism was significantly higher in Group 2 compared to Group 1 (*p* < 0.05) (Table 1).

Similarly, the length of hospital stay and the rate of fibrosis in MRI were significantly higher in the SCD priapism group compared to the non-SCD priapism group (*p* < 0.05) (Table 1). The rate of PP implantation was significantly higher in the SCD priapism group compared to the other priapism groups (*p* < 0.05) (Table 1). There were no significant differences between Group 1 and Group 2 in terms of patient satisfaction, reoperation rates, and postoperative complication rates (*p* > 0.05) (Table 2).

However, the length of hospital stay was significantly longer in the SCD priapism group compared to the other priapism group (*p* < 0.05) (Table 2).

Subgroup analysis showed that there were no statistically significant differences in priapism history, regular hydration therapy, PDE5 inhibitor use, and postoperative complications between patients with SCD-associated priapism who underwent prosthesis implantation and those who did not (*p* > 0.05) (Table 3). The complication rate was similar in the PP implantation and non-PP implantation groups (*p* = 0.292). One patient had a wound infection which was treated medically. However, two patients underwent penile prosthesis extraction due to prosthesis infection and distal perforation.

In the group that underwent PP implantation, the rates of fibrosis in MRI and length of hospital stay were significantly higher than in the group without prosthesis implantation (*p* < 0.05) (Table 3). In addition, 75% of sickle cell patients with priapism who underwent prosthesis implantation had a history of multiple episodes, whereas only 25% of these patients were regular users of hydroxyurea. In contrast, 50% of sickle cell patients with priapism who did not undergo prosthesis implantation had a history of multiple episodes, and 41.7% were regular users of hydroxyurea.

## 4. Discussion

In this study, we compared cases of ischemic priapism secondary to SCD with those caused by other etiologies, based on our single-center experience. Our findings revealed significant differences in the clinical characteristics and management approaches between priapism cases associated with SCD and those attributed to other causes. Notably, ischemic priapism is a prevalent yet often overlooked complication in adult males with SCD [11]. In these patients, failure to promptly recognize priapism, a lack of awareness, or delayed intervention may lead to irreversible tissue damage and erectile dysfunction [12]. Furthermore, early diagnosis and timely management are crucial in preventing long-term erectile dysfunction in this population. In SCD patients, recurrent episodes of brief priapic attacks, frequently occurring during sleep, progress to major priapism in approximately 30% of cases. Several studies have reported that over 70% of patients with a history of severe priapism also have a history of recurrent priapic episodes [13].

The recurrence of priapism in these patients is thought to be associated with nocturnal oxygen desaturation and impaired penile blood flow during sleep, which exacerbate red blood cell sickling and vaso-occlusion in the penile microcirculation. In contrast, in our study, 60% of patients in the SCD group experienced more than one priapism episode, whereas this rate was only 10% in the non-SCD priapism group. Particularly, almost all of the recurrent priapism episodes in the SCD patients occurred during night-time sleep, further strengthening the association between sleep-related hypoxia and priapism in this population. We observed that all SCD patients with a history of recurrent priapism had experienced at least one short-duration (<3 h) priapic episode while sleeping at night. These recurrent nocturnal episodes, commonly seen in SCD patients, are strongly associated with increased nocturnal oxygen desaturation and reduced blood flow, ultimately leading to red blood cell sickling and vaso-occlusion in the penile microvasculature [14]. Such repeated nocturnal episodes heighten the risk of penile tissue damage and permanent erectile dysfunction. Therefore, patient education and the implementation of preventive strategies are essential to mitigating priapism-related complications in individuals with SCD.

Our literature review revealed a lack of definitive evidence regarding the optimal treatment strategy for preventing priapism recurrence in SCD patients [15]. However, potential therapeutic approaches, such as hydroxyurea administration or regular blood transfusions, may be employed for a defined period to assess their effectiveness in reducing the incidence of priapism in at-risk individuals [16]. Hydroxyurea, which became the first FDA-approved drug for SCD treatment in 1998, has demonstrated significant benefits in decreasing the frequency and severity of vaso-occlusive crises while improving the overall quality of life in SCD patients [17]. Nevertheless, an animal study reported that hydroxyurea treatment did not modify the priapism phenotype in transgenic SCD mice, suggesting that its efficacy in preventing priapism via hemolysis reduction may be limited [18]. Our study noted low compliance with hydroxyurea therapy among SCD patients with priapism, which may contribute to the high recurrence rates of priapism in this cohort. Specifically, only 35% of SCD patients (7 out of 20) in our study were consistently using hydroxyurea. When comparing SCD patients with and without penile prostheses, the use of hydroxyurea was lower in the prosthesis group than in the non-prosthesis group (25% vs. 41.7%), although this difference was not statistically significant. These findings suggest that the effectiveness of hydroxyurea or other therapeutic options in preventing priapism warrants further exploration in larger, long-term studies.

The timing of presentation to the hospital is another critical factor that significantly influences clinical outcomes in priapism patients. Early medical intervention is especially vital in cases of ischemic priapism to prevent irreversible tissue damage. Although there is a limited body of literature on the average time of presentation for priapism patients, the existing data indicate that delayed presentation negatively affects treatment outcomes and increases the risk of complications [19]. Physiological changes and microscopic tissue damage in the penis during priapism typically begin within 6 h of onset. After 24 h, cellular damage, increased platelet aggregation, and the destruction of sinusoidal endothelial cells become more pronounced; by 36 h, thrombus formation and cavernosal tissue damage lead to fibrosis and up to 90% permanent erectile dysfunction [20]. In this study, the average time to presentation was 24 h in Group 1 and 48 h in Group 2. MRI imaging showed fibrosis in 65% of Group 2 patients (13 out of 20), whereas Group 1 had no fibrosis.

Ultrasonography can also be valuable in assessing penile fibrosis, with elastography emerging as a particularly promising tool for its detection. A study by Hamidi et al. demonstrated that patients with ED following radical prostatectomy exhibited lower elasticity scores, leading the authors to suggest that penile elastography could serve as a predictive tool for detecting penile fibrosis [21]. Consistent with these findings, a recent study reported that shear wave elastography was superior in identifying early histological changes in corporal cavernosal fibrosis [22]. In our study, however, we were only able to detect the ischemic pattern in all participants. These data suggest that delayed presentation to the hospital may be statistically significant in its association with the development of fibrosis. Specifically, delayed presentation increases the risk of fibrosis and subsequent permanent tissue damage. Early medical intervention is crucial in preventing irreversible tissue damage and long-term complications, such as permanent erectile dysfunction, in priapism patients. In this context, the observation of fibrotic changes resulting from delayed intervention underscores the critical importance of timely and appropriate management in priapism. This finding indicates that the pathological fibrotic process, if left untreated, can lead to irreversible tissue damage and subsequent functional loss, thus highlighting the necessity for strategic management regarding the timing of treatment protocols.

Sickle cell disease is associated with an increased risk of erectile dysfunction due to penile fibrosis. However, other mechanisms, such as the downregulation of the NO/cGMP/PKG signaling pathway, reduced PDE5 regulatory function in the penis, and uncontrolled vasorelaxation of the cavernous smooth muscle, can also contribute to the development of priapism [23]. These mechanisms suggest that patients may experience erectile dysfunction before the development of penile fibrosis. In our study, patients with ED had lower scores in the SCD group compared to the non-SCD group. However, we attribute these findings to the small sample size and the slightly younger age of patients in the SCD group.

As clearly indicated in the literature, delayed patient presentation significantly diminishes the effectiveness of first-line treatment methods, increasing the need for PP implantation and promoting the necessity of surgical intervention [7]. In our study, all the patients in Group 1 responded well to first-line treatments (corporal aspiration or saline irrigation) and reported no persistent erectile dysfunction. However, in Group 2, the rate of PP implantation was significantly higher, at 40% (8 out of 20 patients), compared to Group 1. All patients who underwent prosthesis implantation were operated on early (within the first week). The effectiveness of first-line treatment methods in Group 1 was further supported by the absence of persistent erectile dysfunction. In contrast, the high rate of PP implantation in Group 2 indicates that the treatment strategy for this group was more invasive.

Although PP implantation is a common treatment option for delayed ischemic priapism, the literature is limited regarding the optimal timing of surgery, types of prosthesis, and associated complications. Early PP implantation facilitates a quicker return to sexual activity and prevents penile shortening due to corporal fibrosis. However, it may also be associated with risks such as edema, infection, and distal perforation, particularly in patients undergoing shunt procedures [24].

The most frequently reported complications following PP implantation procedures in the literature include edema and wound infection (6–50%), erosion (1–9%), and mechanical issues related to the prosthesis (2–5%) [25]. Consistent with the literature, patients with SCD who underwent PP implantation had a similar complication rate. However, two patients required prosthesis extraction due to complications. We believe that SCD patients are more prone to complications after prosthesis surgery due to vascular dysfunction and poor tissue oxygenation.

The absence of serious early complications in patients who underwent PP implantation suggests the reliability of this treatment method. The lack of a significant difference in complication rates between the two groups, as well as between patients with and without prostheses, indicates that the short-term safety profiles of the treatment options are comparable. However, a definitive conclusion cannot be drawn without long-term efficacy and safety evaluations. To fully understand the long-term efficacy of PP implantation and conservative management, comprehensive and prolonged analyses are necessary to assess their durability, complication rates, patient satisfaction, and impact on quality of life. Such analyses will enable more definitive and reliable conclusions regarding the sustainability of these treatments and their potential effects on patients with priapism. On the other hand, a significant proportion of individuals with SCD, despite experiencing priapistic episodes, tend not to seek medical attention. A survey conducted by Tanabe and colleagues found that only 7% of SCD patients were aware of priapism and its potential complications [26]. This finding suggests that SCD patients have a limited awareness of the serious complications associated with priapism and the importance of timely medical intervention. In our study, data regarding the awareness of priapism and potential urological complications among the SCD patient group was not collected. This limitation represents a methodological shortcoming of our study. However, the late presentation of SCD patients to healthcare facilities, the high rate of PP implantation in this group, and the low rate of regular hydroxyurea use support the notion that this patient group may have an insufficient awareness of priapism and its potential complications.

The limited number of patients included in this study may present certain limitations on the generalizability of the results. While our study presents significant findings in comparing ischemic priapism caused by SCD with ischemic priapism resulting from other etiologies, the results derived from a small patient cohort should be validated in larger-scale studies. In this regard, the low patient number may limit the statistical power of the findings, which is an important factor to consider when interpreting the results. Future studies involving larger patient populations could enhance the validity and generalizability of our findings.

Another important limitation is the lack of data regarding long-term patient satisfaction, prosthesis durability, and postoperative complications. As our study is retrospective in nature, follow-up data were collected from medical records, and the available follow-up duration varied among patients. However, to enhance clarity, we have specified the early follow-up period in the manuscript. While detecting penile fibrosis using MRI is a valuable tool, penile elastography could have provided additional insights into the extent of fibrosis and tissue changes in priapism cases. Furthermore, we acknowledge that the inability to perform elastography constitutes a limitation of our study.

## 5. Conclusions

Ischemic priapism associated with SCD represents a significant clinical issue, particularly among males living in endemic regions. This study has demonstrated that patients with SCD experience more frequent recurrent priapism episodes, present to the hospital later, and, consequently, have a significantly increased requirement for PP implantation compared to patients with other causes of ischemic priapism. The high incidence of recurrent priapism in SCD patients and their delayed hospital presentation underline the need for improved awareness and early intervention in this population. Additionally, the low level of priapism awareness among individuals with SCD contributes to their delayed presentation and an elevated risk of permanent erectile dysfunction. Educating both patients and healthcare providers about the early signs and risks of priapism is essential in reducing the long-term complications of this condition. Hematologists and urologists play a critical role in raising awareness among patients and ensuring early intervention. More detailed and advanced research on the management of SCD-associated priapism could improve the effectiveness of current treatment approaches and enhance patient outcomes. Future studies with larger patient cohorts are needed to establish standardized treatment protocols and assess the long-term efficacy of various management strategies in preventing priapism recurrence and minimizing erectile dysfunction in SCD patients.

## Figures and Tables

**Table 1:** General characteristics of patients with SCD and other priapism.

		Other Priapism		SCD Priapism		*p*
		Average ± ss/n (%)	Median	Average ± ss/n (%)	Median	
Age		44.0 ± 12.9	42.5	38.0 ± 14.2	40.0	0.167 ^t^
Comorbidity	(−)	18 (90.0%)		0 (0.0%)		**0.000** X^2^
	(+)	2 (10.0%)		20 (100.0%)		
History of priapizm	(−)	18 (90.0%)		8 (40.0%)		**0.001** X^2^
	(+)	2 (10.0%)		12 (60.0%)		
Aspiration before PPI	(−)	1 (5.0%)		2 (10.0%)		1.000 X^2^
	(+)	19 (95.0%)		18 (90.0%)		
History of ED	(−)	7 (35.0%)		11 (55.0%)		0.204 X^2^
	(+)	13 (65.0%)		9 (45.0%)		
Time to hospital presentation (days)		0.9 ± 0.4	1.0	2.4 ± 1.6	2.0	**0.000** ^m^
**MR**						
Normal		20 (100.0%)		7 (35.0%)		**0.000** X^2^
Fibrosis		0 (0.0%)		2 (10.0%)		
Dense fibrosis		0 (0.0%)		11 (55.0%)		
Prosthesis	Not placed	20 (100.0%)		12 (60.0%)		**0.002** X^2^
	Placed	0 (0.0%)		8 (40.0%)		
Hgb		13.9 ± 1.3	14.0	8.0 ± 0.7	8.0	**0.000** ^t^
Hct		42.2 ± 3.4	42.6	23.5 ± 3.2	24.2	**0.000** ^t^
Blood gas pH		7.1 ± 0.1	7.1	6.9 ± 0.1	7.0	**0.000** ^m^

^t^ indicates independent sample *t* test; ^m^ indicates Mann–Whitney U test; X^2^ indicates chi-square test (Fischer’s test); SCD: sickle cell disease.

**Table 2:** Complications and patient satisfaction in patients with SCD and other priapism.

		Other Priapism		SCD Priapism		*p*
		Average ± ss/n (%)	Median	Average ± ss/n (%)	Median	
Postoperative complication	(−)	19 (95.0%)		15 (75.0%)		0.077 X^2^
	(+)	1 (5.0%)		5 (25.0%)		
***Antibiotic***						
Ciprofloxacin		2 (10.0%)		0 (0.0%)		0.487 X^2^
Zygosis		0 (0.0%)		4 (20.0%)		**0.035** X^2^
Candisept		0 (0.0%)		4 (20.0%)		**0.035** X^2^
Ceftriaxone		5 (25.0%)		9 (45.0%)		0.185 X^2^
Cefazolin		13 (65.0%)		5 (25.0%)		**0.011** X^2^
Meronem		0 (0.0%)		5 (25.0%)		**0.017** X^2^
Vancomycin		0 (0.0%)		8 (40.0%)		**0.002** X^2^
Tazocin		0 (0.0%)		8 (40.0%)		**0.002** X^2^
Linezolid		0 (0.0%)		1 (5.0%)		1.000 X^2^
Fluconazole		0 (0.0%)		4 (20.0%)		**0.035** X^2^
Hospitalization time (day)		1.2 ± 0.7	1.0	5.9 ± 8.3	2.5	**0.003** ^m^
***Treatment satisfaction***						
Not satisfied		0 (0.0%)		4 (20.0%)		0.105 X^2^
Satisfied		13 (65.0%)		11 (55.0%)		
Highly satisfied		7 (35.0%)		5 (25.0%)		

^m^ indicates Mann–Whitney U test; X^2^ indicates chi-square test (Fischer’s test).

**Table 3:** General characteristics of patients with and without prosthesis implantation in SCD patients.

			Prosthesis Placement (−)		Prosthesis Placement (+)		*p*
			Average ± ss/n (%)	Median	Average ± ss/n (%)	Median	
**Age**			**41.4 ± 16.8**	**43.0**	32.8 ± 7.2	30.5	0.188 ^t^
History of priapism	(−)		6 (50.0%)		2 (25.0%)		0.264 X^2^
	(+)		6 (50.0%)		6 (75.0%)		
Regular hydroxyurea therapy	(−)		7 (58.3%)		6 (75.0%)		0.444 X^2^
	(+)		5 (41.7%)		2 (25.0%)		
Aspiration before PPI	(−)		2 (16.7%)		0 (0.0%)		0.495 X^2^
	(+)		10 (83.3%)		8 (100.0%)		
History of ED	(−)		8 (66.7%)		3 (37.5%)		0.199 X^2^
	(+)		4 (33.3%)		5 (62.5%)		
Time to hospital admission (day)			1.6 ± 1.0	2.0	3.5 ± 1.6	3.0	**0.004** ^m^
***MR***							
Normal			7 (58.3%)		0 (0.0%)		**0.007** X^2^
Fibrosis			2 (16.7%)		0 (0.0%)		
Dense fibrosis			3 (25.0%)		8 (100.0%)		
Postoperative complication		(−)	10 (83.3%)		5 (62.5%)		0.292 X^2^
		(+)	2 (16.7%)		3 (37.5%)		
Hospitalization time (day)			1.7 ± 1.3	1.0	12.1 ± 10.6	7.0	**0.000** ^m^
***Patient satisfaction***							
Not satisfied			3 (25.0%)		1 (12.5%)		0.619 X^2^
Satisfied			8 (66.7%)		3 (37.5%)		
Highly satisfied			1 (8.3%)		4 (50.0%)		

^t^ indicates independent sample *t* test; ^m^ indicates Mann–Whitney U test; X^2^ indicates chi-square test (Fischer’s test).

## Data Availability

Our study data are unavailable due to privacy and ethical restrictions.
